# Draft Genome Sequence of Pediococcus pentosaceus IMI 507024

**DOI:** 10.1128/mra.01216-21

**Published:** 2022-03-28

**Authors:** Ivana Nikodinoska, Jenny Makkonen, Daniel Blande, Colm Moran

**Affiliations:** a Alltech European Headquarters, Sarney, Dunboyne, Co. Meath, Ireland; b Biosafe – Biological Safety Solutions Ltd., Kuopio, Finland; c Regulatory Affairs Department, Alltech SARL, Vire, France; Indiana University, Bloomington

## Abstract

We report here the draft genome sequence of Pediococcus pentosaceus strain IMI 507024, a lactic acid bacterium isolated from fermented sausage in Kentucky (Nicholasville, KY, USA). The strain is deposited in the Centre for Agriculture and Bioscience International (CABI) Culture Collection with the accession number IMI 507024.

## ANNOUNCEMENT

Preservation of forage material for animal nutrition use is an ongoing challenge. Microbial inoculants such as *Pediococcus* spp. may play an important role during silage production via fermentation loss inhibition and nutritional quality improvement (1–3). This study describes the whole-genome sequence of Pediococcus pentosaceus IMI 507024.

A single colony was obtained on DeMan, Rogosa, and Sharpe (MRS) agar and incubated anaerobically at 37°C for 48 h. For genome analyses, a colony was transferred to 10 mL MRS broth and incubated at 37°C under anaerobic conditions for 16 to 17 h. Genomic DNA was extracted according to the sample preparation and lysis protocol described for Gram-negative and some Gram-positive bacterial samples in the Qiagen Genomic DNA Handbook (Qiagen) and purified according to the Genomic-tip 100/G (Qiagen) procedure. DNA was submitted to Eurofins Genomics (Constance, Germany) for library preparation (NEBNext Ultra II FS DNA library prep kit; New England Biolabs, San Diego, CA, USA) and whole-genome sequencing (NovaSeq 6000; Illumina), producing 2 × 150-bp paired-end reads. The sequencing produced 6,569,830 raw reads. The reads were trimmed using Trimmomatic v0.38.1 with the settings ILLUMINACLIP 2:30:10:8 and SLIDINGWINDOW:4:20 (4) and assembled using Unicycler v0.4.8 (5), generating 17 contigs with a genome length of ∼1.80 Mb, 1,013-fold coverage, GC content of 37.03%, and *N*_50_ value of 354,566 bp. The annotation was performed with the NCBI Prokaryotic Genome Annotation Pipeline (PGAP) v6.0 (6), obtaining an annotated assembly of 12 contigs, 1,812 genes, 1,752 of which were coding sequences (CDS), 3 rRNAs, and 47 tRNAs. The IMI 507024 genome showed a Mash (MinHash v0.1.1 [7]) distance of 0.00671909 and a calculated OrthoANI (OrthoANI v1.40 [8]) value of 98.88% with P. pentosaceus ATCC 25745. The average nucleotide identity (ANI) value with the P. pentosaceus type strain ATCC 33316 (GenBank accession number PUFB00000000) was 99.62%.

Searches for antimicrobial resistance genes were made against the NCBI Bacterial Antimicrobial Resistance Reference Gene database (NCBI BioProject number PRJNA313047, database v2021-06-01.1), using AMRFinderPlus v3.10.5 (9) and DIAMOND (Galaxy v0.9.29.0 [10]). AMRFinderPlus was run in combined mode performing searches on genome and predicted protein sequences. Parameters set for this search included minimum identity of 45% and minimum coverage of 25%. Searches were also performed against the ResFinder database (11) (downloaded on 20 April 2021) using blastn. Default parameters were used for all software except where otherwise noted. No acquired antimicrobial resistance genes of concern that were above 80% identity and 70% coverage (12) were found.

Toxin and virulence factor genes potentially carried by IMI 507024 were screened using DIAMOND v2.0.8 (settings mode blastp –more-sensitive --matrix ‘BLOSUM62’ --comp-based-stats ‘1’ --masking ‘1’ --evalue ‘0.01’ --id ‘80’ --query-cover ‘0’ --subject-cover ‘0’) (13, 14). No genes encoding potential virulence or pathogenicity factors were identified.

Plasmids were searched from the genome data by screening the contigs against the PlasmidFinder database (15). Assembly files were examined for circular contigs, and Blast searches were conducted to identify if contigs were likely to be plasmids. No plasmid-related sequences matched against the PlasmidFinder database.

### Data availability.

All data are available under BioProject accession number PRJNA777113, SRA accession number SRP344187, and GenBank accession number JAJFOC000000000.2.
